# Recent advances of engineering cell membranes for nanomedicine delivery across the blood–brain barrier

**DOI:** 10.1186/s12951-025-03572-y

**Published:** 2025-07-08

**Authors:** Shengnan Yuan, Dehong Hu, Duyang Gao, Christopher J. Butch, Yiqing Wang, Hairong Zheng, Zonghai Sheng

**Affiliations:** 1 Research Center for Advanced Detection Materials and Medical Imaging Devices, Institute of Biomedical and Health Engineering, Shenzhen Institute of Advanced Technology, Chinese Academy of Sciences, Shenzhen, 518055 P. R. China; 2 State Key Laboratory of Biomedical Imaging Science and System, Shenzhen, 518055 P. R. China; 3 Department of Biomedical Engineering, College of Engineering and Applied Sciences, State Key Laboratory of Analytical Chemistry for Life Science, Nanjing University, Nanjing, 210023 P. R. China

**Keywords:** Blood-brain barrier, Cell membrane-engineered nanocarriers, Central nervous system disorder, Clinical translation, Artificial intelligence

## Abstract

The blood-brain barrier (BBB) poses a major challenge to the effective delivery of therapeutic agents for the treatment of central nervous system (CNS) disorders. The integration of cell membrane engineering with nanotechnology has recently enabled the development of membrane-engineered nanoparticles (CNPs). These nanocarriers exhibit enhanced BBB penetration and improved CNS targeting. This review systematically summarizes the latest advances in the development and application of CNPs, emphasizing how different cellular sources—such as erythrocytes, platelets, tumor cells, and leukocytes—impact delivery efficiency and therapeutic outcomes. We also examine the molecular mechanisms underlying nanoparticle-BBB interactions and highlight the importance of biosafety evaluations. Moreover, critical barriers to clinical translation, including large-scale manufacturing challenges, batch-to-batch variability, and regulatory complexities, are discussed. Finally, we explore emerging strategies—particularly the integration of artificial intelligence (AI)—that hold potential for overcoming existing clinical gaps, enabling the rational design and optimized development of CNP-based therapeutics for CNS disorders. By integrating mechanistic insights with translational perspectives, this review provides a clear conceptual and technological foundation for the development of next-generation CNS-targeted nanotherapeutics.

## Graphical Abstract

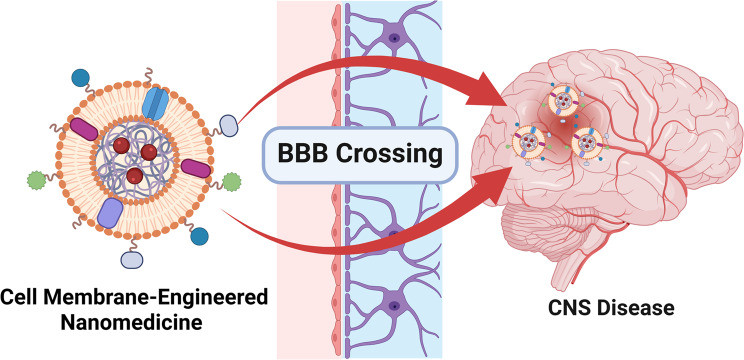

## Introduction

**Fig. 1:**
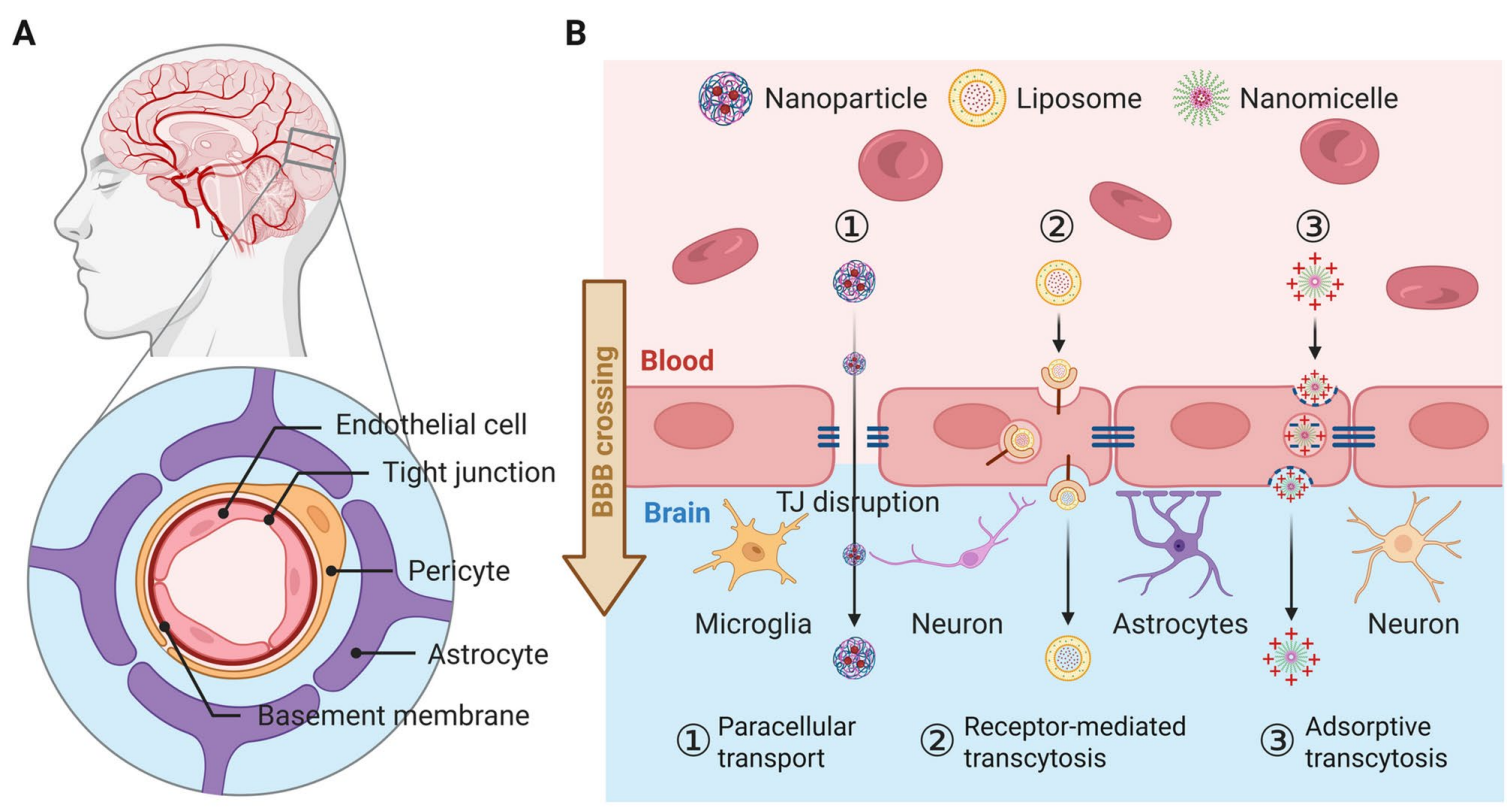
**(A)** Schematic illustration of the BBB structure. **(B)** Diagram depicting the passive transcytosis mechanisms of nanomedicines across the BBB. Abbreviations: TJ, tight junction. The image was created using BioRender.com and is used with permission

The treatment of central nervous system (CNS) disorders—encompassing neurodegenerative diseases, cerebrovascular accidents, and primary malignant brain tumors—remains one of the most formidable challenges in contemporary medicine [1]. Although pharmacotherapy plays a critical role in managing CNS pathologies, its efficacy is severely hindered by the limited transport of most therapeutic agents across the blood-brain barrier (BBB). As a highly specialized physiological interface, the BBB tightly regulates molecular transport into the brain, often resulting in the exclusion of potentially neurotherapeutic agents. Structurally, the BBB is composed of three key components: brain microvascular endothelial cells interconnected by tight junctions, pericytes embedded within the vascular basement membrane, and astrocytic end-feet forming the neurovascular unit (Fig. 1A). This intricate architecture blocks the entry of over 98% of potential neuropharmaceuticals, including biomacromolecules, antibodies and nanoparticle-based therapeutics, severely limiting their therapeutic potential [1–3]. Physical strategies such as focused ultrasound [4–6] and photothermal irradiation [7–9] have been demonstrated to enhance BBB drug permeability by transiently compromising the integrity of endothelial tight junctions, but these approaches are limited by requirement of specialized equipment and risk of infection or tissue damage [2]. Thus, there remains a pressing need for efficient, safe, and noninvasive strategies to overcome the BBB for CNS-targeted therapy.

Since the Food and Drug Administration (FDA) approval of PEGylated liposomal doxorubicin (Doxil^®^) in 1995 [10], nanotechnology has revolutionized drug delivery. This milestone catalyzed the development of advanced nanocarriers, including polymeric nanoparticles, dendrimers, and micelles [11, 12]. To improve BBB transcytosis, ligand-functionalized nanoparticles targeting endothelial surface receptors (e.g., transferrin receptor, insulin receptor, and low-density lipoprotein receptor-related protein [LRP]) have shown promising results in preclinical glioma models [13, 14]. In addition, surface modification with cationic peptides facilitates adsorptive-mediated transcytosis, thereby enhancing nanoparticle penetration (Fig. 1B). Despite significant advances in pharmacokinetics, controlled release, and reduced off-target toxicity, the clinical translation of these nanoplatforms remains limited by rapid clearance via the mononuclear phagocyte system (MPS) and potential immunogenicity [15–17]. For CNS delivery, the dual challenge of evading immune recognition while achieving effective BBB penetration underscores the limitations of current approaches. To address these barriers, Zhang et al. introduced a transformative concept in 2011—cell membrane-engineered nanoparticles (CNPs) [18]. This biomimetic strategy leverages the natural trafficking capabilities of endogenous cells by cloaking synthetic nanoparticle cores with plasma membranes derived from erythrocytes, leukocytes, or stem cells. The resulting CNPs inherit essential biological functions, including immune evasion, tissue-specific targeting, and enhanced transendothelial migration. By improving systemic circulation time and BBB permeability, this approach has significantly advanced the development of CNS-targeted nanotherapeutics [19–21].

In this review, we provide a comprehensive overview of membrane-engineered nanoparticles for CNS drug delivery. We discuss the biological rationale, design strategies, and mechanistic insights underlying BBB translocation. Special attention is given to the delivery performance of CNPs derived from various cellular sources, as well as the challenges impeding their clinical translation. We conclude by highlighting emerging technologies—including artificial intelligence—that may accelerate the development of next-generation nanomedicines for the diagnosis and treatment of CNS disorders.

## CNP fabrication methods

**Fig. 2:**
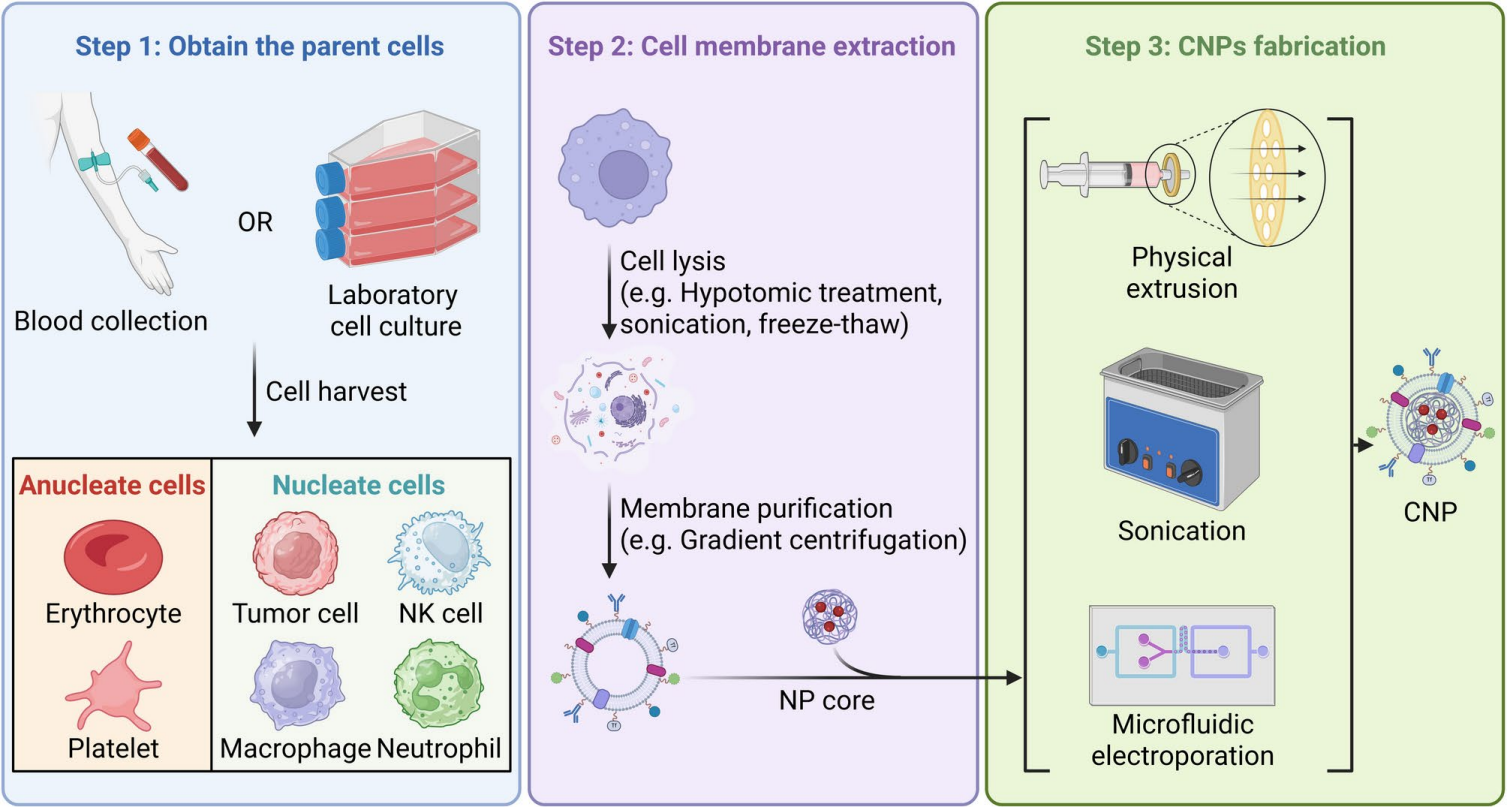
Fabrication methods of cell membrane-coated nanoparticles (CNPs). Parent cells are first collected and subjected to membrane extraction. The isolated cell membranes are subsequently coated onto the nanoparticle (NP) core using one of three different techniques: physical extrusion, sonication, or microfluidic electroporation. The image was created using BioRender.com and is used with permission

Fabrication of CNPs is the process of hybridizing natural cellular membrane components onto a synthetic core. This process is initiated by obtaining and purifying the source cells (Fig. 2). Erythrocytes, platelets, and leukocytes (e.g., macrophages, neutrophils, and natural killer [NK] cells) can be readily harvested from peripheral blood through standard processing techniques, offering a cost-effective and straightforward approach [19]. Alternatively, laboratory-cultured blood cells are also feasible and have been utilized when membrane modifications are required [22]. For cancer cell membrane-camouflaged CNPs, stable cell lines maintained under moderate-scale laboratory conditions are typically used [19], while ongoing studies are also exploring the use of membranes derived from resected primary tumor tissues to support the development of personalized drug delivery systems [23]. Membrane materials are then isolated from other cellular components, typically by physical disruption and mechanical separation. Due to the anucleate nature and simple intracellular architecture of erythrocytes and platelets, membranes from these cells can be efficiently obtained via repeated freeze-thaw cycles followed by high-speed centrifugation. In contrast, membrane extraction from nucleated cells—such as tumor cells, macrophages, NK cells, and neutrophils—requires gradient centrifugation following cell lysis and homogenization [19].

After purification, the resulting membrane vesicles are employed to decorate nanoparticle cores using techniques such as physical extrusion, ultrasonication, or microfluidic mixing. Physical extrusion is preferred when membrane integrity must be preserved, as it involves repeatedly co-extruding membrane vesicles and nanoparticle cores through porous membranes to achieve uniform coating [18, 24]. Extrusion also effectively preserves the correct display of functional proteins on the NP surface, thereby retaining biological activity [19]. Ultrasonication applies ultrasonic energy to efficiently facilitate membrane-nanoparticle fusion [25], though care must be taken not to damage membrane structural integrity or function of embedded proteins through excessive exposure [19]. Microfluidic systems enable the controlled assembly of membrane-coated nanoparticles within narrow reaction channels, offering improved heat and mass transfer as well as precise mixing. Similar to ultrasonic treatment, high microfluidic shear stresses can deform membranes and denature surface proteins, making careful optimization of microfluidic parameters essential to minimize structural disruption and maintain membrane functionality [26].

Currently, the fabrication of CNPs is primarily confined to laboratory-scale protocols, while large-scale production remains a significant challenge due to technological limitations. Even in small-scale production, precise optimization of fabrication parameters is essential to overcome significant batch-to-batch variability. To address these issues, rigorous quality assessments of the coated particles (e.g., evaluations of coating stability, membrane integrity, and successful membrane fusion) are necessary to ensure reproducibility and functional consistency [27].

## BBB crossing mechanisms of CNPs

**Fig. 3:**
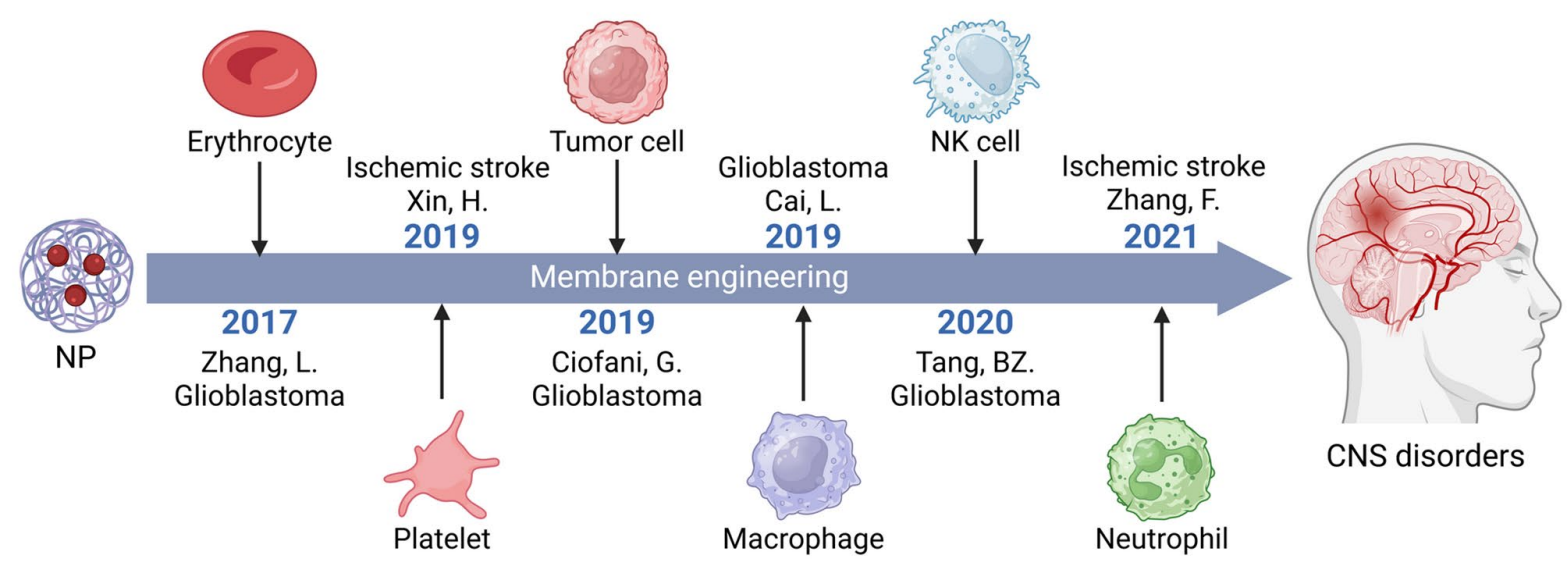
Development timeline of CNPs for BBB-crossing drug delivery. The image was created using BioRender.com and is used with permission

Due to the unique characteristics of biologically sourced membranes, CNP-based drug delivery systems derived from different parent cells exhibit significant advantages and deficiencies in circulation time, immune evasion, and targeting specificity [28] (Table 1). Additionally, the membrane proteins inherited from these cells contribute to cell-specific mechanisms of BBB penetration and disease targeting. A development timeline of various CNPs beginning with erythrocyte-derived CNPs, including platelets, brain tumor cells, macrophages, NK cells, and neutrophils, is depicted in Fig. 3, while Table 1 contains a summary description of the unique strengths and weaknesses in BBB penetration of CNPs derived from these cells. In the following subsections, we highlight the mechanistic insights underlying the unique brain-targeting characteristics of each of these CNP types for drug transport across the BBB.

**Table 1:** Advantages and deficiencies of CNPs derived from various parent cell types

Parent cell	Advantages of CNPs	Deficiencies of CNPs
Erythrocyte	Prolonged circulation time Immune evasion Easy membrane extraction Capability to neutralize membrane attack toxins	Poor targeting specificity
Platelet	Immune evasion Straightforward extraction Active participation in hemostasis, thrombosis, and inflammation Tumor-targeting ability	Short circulation time
Tumor cell	Homotypic tumor targeting Antigen presentation and immune activation	Difficult extraction Limited circulation time
Leukocyte	Inflammation-site accumulation Tumor-homing capacity	Challenging extraction Limited circulation time

### BBB crossing mechanisms of Erythrocyte-derived CNPs

**Fig. 4:**
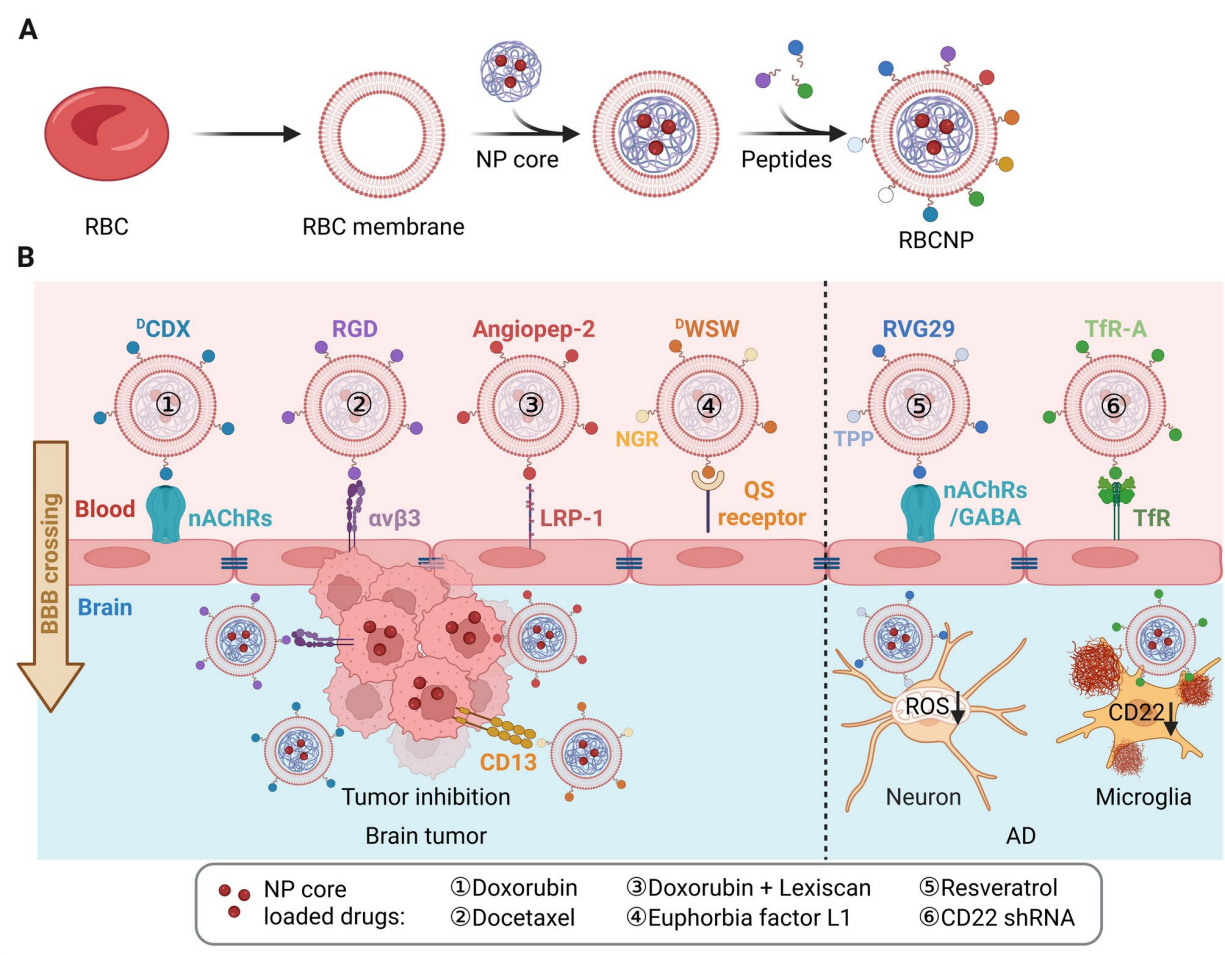
**(A)** Schematic illustration of erythrocyte membrane-engineered nanoparticle (RBCNP) fabrication, illustrating the integration of various peptides onto the RBCNP surface. **(B)** Mechanisms of RBCNP-mediated BBB crossing, facilitated by peptide modifications and interactions with corresponding endothelial cell membrane receptors. RBCNPs have been utilized for the treatment of brain tumors and Alzheimer’s disease (AD). The image was created using BioRender.com and is used with permission

Erythrocyte or red blood cell membrane-engineered nanoparticles (RBCNPs) exhibit extended systemic circulation and immune evasion due to the presence of the “self-marker” CD47, which binds to the signal regulatory protein-α (SIRP-α) on macrophages, thereby inhibiting phagocytosis. In comparison with conventional encapsulation techniques, such as physical encapsulation strategies, RBCNPs demonstrated superior control over drug release kinetics, primarily attributed to the synergistic effects of their biomimetic membrane coating. The erythrocyte membrane cloak acted as a diffusion barrier, retarding outward drug efflux [29]. As erythrocyte membranes are easily obtainable, RBCNP-based nanocarriers were among the first evaluated for CNS drug delivery, particularly for glioma [30–33] and AD treatment [34–36]. However, their intrinsic BBB permeability and disease-targeting capacity are limited, necessitating further ligand modification (Fig. 4).

In 2017, Zhang et al. introduced a BBB-penetrating glioma nanotherapy using RBCNPs modified with the ^D^CDX peptide, which targets nicotinic acetylcholine receptors (nAChRs) on brain endothelial cells to mediate receptor-driven transcytosis. Noticeably, a streptavidin–biotin insertion strategy was adopted to preserve ligand function, as the traditional lipid insertion may cause electrostatic interference between positively charged ligands and the negatively charged membrane. Compared to unmodified controls, a 2-fold increase in transcytosis was observed in primary brain endothelial cells treated with ^D^CDX-RBCNPs. In vivo, ^D^CDX-RBCNPs localized predominantly at cortical tumor sites, while unmodified RBCNPs remained confined to the vasculature. These results indicated a significantly enhanced BBB penetration of RBCNPs upon ^D^CDX modification [30]. Similarly, RBC nanocrystals modified with c(RGDyK) peptide (RGD-RBCNCs) significantly enhanced docetaxel (DTX) delivery to glioma by interacting with integrin αvβ3, both on the surface of endothelial and tumor cells. In murine models, brain accumulation of DTX at 2 and 24 h post-injection was significantly higher when carried by RGD-RBCNCs in comparison with unmodified RBCNCs [32]. Additionally, by targeting LRP1 on the surface of brain endothelial cells, Shi et al. designed an angiopep-2 peptide-functionalized RBCNP (Ang-RBC@NM) for Dox delivery across BBB. Furthermore, the co-loading of Lexiscan (Lex), an adenosine A2A adenosine receptor agonist, transiently disrupted BBB and further enhanced the BBB penetration of the nanoparticles. As a result, the orthotopic brain tumor accumulation of Ang-RBC@NM-(Dox/Lex) was 21.9- and 2.5-fold higher than the free Dox controls and RBC@NM-(Dox/Lex), respectively. Glioma-bearing mice injected with Ang-RBC@NM-(Dox/Lex) also showed the best survival rate among free Dox and RBC@NM-(Dox/Lex) [31].

Dual-ligand modification has further improved RBCNP performance. For instance, RBCNPs modified with both ^D^WSW, a BBB-crossing peptide interacting with the quorum-sensing (QS) receptor, and NGR, a glioma cell recognizing peptide specifically binding to CD13 receptor, exhibited enhanced brain-tumor targeting Euphorbia factor L1 (EFL1) delivery. The significantly increased brain and tumor-site accumulation of dual-modified RBCNPs was demonstrated compared to mono-modified or unmodified controls. Furthermore, the median survival time of glioma-bearing mice also increased to 36 days when treated with ^D^WSW/NGR-RBCNPs, in comparison to 30, 28, and 24 days in those treated with ^D^WSW-, NGR-, and unmodified RBCNPs, respectively [33]. These findings validate that dual-peptide modification significantly promotes the trans-BBB drug delivery efficiency of RBCNPs while concurrently enhancing their targeting specificity toward brain tumors.

Beyond glioma, RBCNPs have also been explored for AD therapy. Resveratrol (RSV), an antioxidant with neuroprotective properties, was delivered to neurons using RBCNPs dual-modified with RVG29 and triphenylphosphonium (TPP), facilitating mitochondrial targeting and reducing oxidative stress in AD mouse models [34]. However, the precise BBB-crossing pathway of RVG29 remains controversial. While some studies indicate that RVG29 interacts with nAChRs on brain endothelial cells to facilitate BBB penetration [37], other findings suggest that it may instead mediate BBB crossing via the GABA(B) receptor [38]. In addition to chemical reagents, RBCNPs were also investigated for genetic therapies. RBCNPs modified with transferrin receptor aptamer (TfR-A) were employed for brain-targeted CD22 shRNA plasmid delivery, inhibiting abnormal CD22 expression in aging microglia and enhancing Aβ phagocytosis in AD mouse brain tissue [35]. The brain accumulation of TR-RBCNPs was 2.0- and 1.24-fold higher than free nanoparticles and unmodified RBCNPs, respectively, indicating the critical role of the TfR aptamer in BBB penetration. The Morris water maze assay further demonstrated that TR-RBCNP-treated mice exhibited significantly improved memory function compared to other treatment groups. Despite the insufficiency of current studies, the application of erythrocyte membrane engineering for the treatment of neurodegenerative diseases such as AD has shown considerable outcomes in preclinical studies, representing a promising avenue for further investigations.

In vivo biosafety studies across multiple RBCNP formulations have shown reduced toxicity compared to free drugs and uncoated nanoparticles. Mice treated with free Dox or DTX displayed significant weight loss, cardiac and renal toxicity [30–32]. In contrast, RBCNP formulations—including ^D^CDX-RBCNP-(Dox) [30], Ang-RBCNP-(Dox/Lex) [31], RGD-RBCNC-(Dox) [32], ^D^WSW/NGR-RBCNP-(EFL1) [33], TfR-RBCNP-(RNAi) [35], and RVG29/TPP-RBCNP-(RSV) [34]—did not induce observable organ damage. Reported plasma half-lives—including ^D^CDX-RBCNP (33.3 ± 4.1 h), Ang-RBCNP (9.3 h), RGD-RBCNC (19.7 ± 1.1 h), and RVG29/TPP-RBCNP (8.06 ± 0.39 h)— demonstrated the long circulation lifespan of RBCNPs, which was not affected by the peptide modifications. Biodistribution studies further confirmed the predominant accumulation in the liver and spleen, suggesting that these organs are the primary sites of metabolism for RBCNPs [33, 35, 36].

Collectively, these findings highlight RBCNPs as a promising platform for brain-targeted drug delivery in glioma, AD, and potentially for other CNS diseases in the future. With the advantages of long circulation time and being easy to obtain, the insufficient brain- and tumor-targeting ability of RBCNPs is also obvious. Therefore, peptide modification is crucial for enhancing BBB permeability and tumor-targeting specificity. Nevertheless, consistent biosafety across diverse formulations underscores the translational potential of RBCNPs for future clinical applications.

### BBB crossing mechanisms of Platelet-derived CNPs

**Fig. 5:**
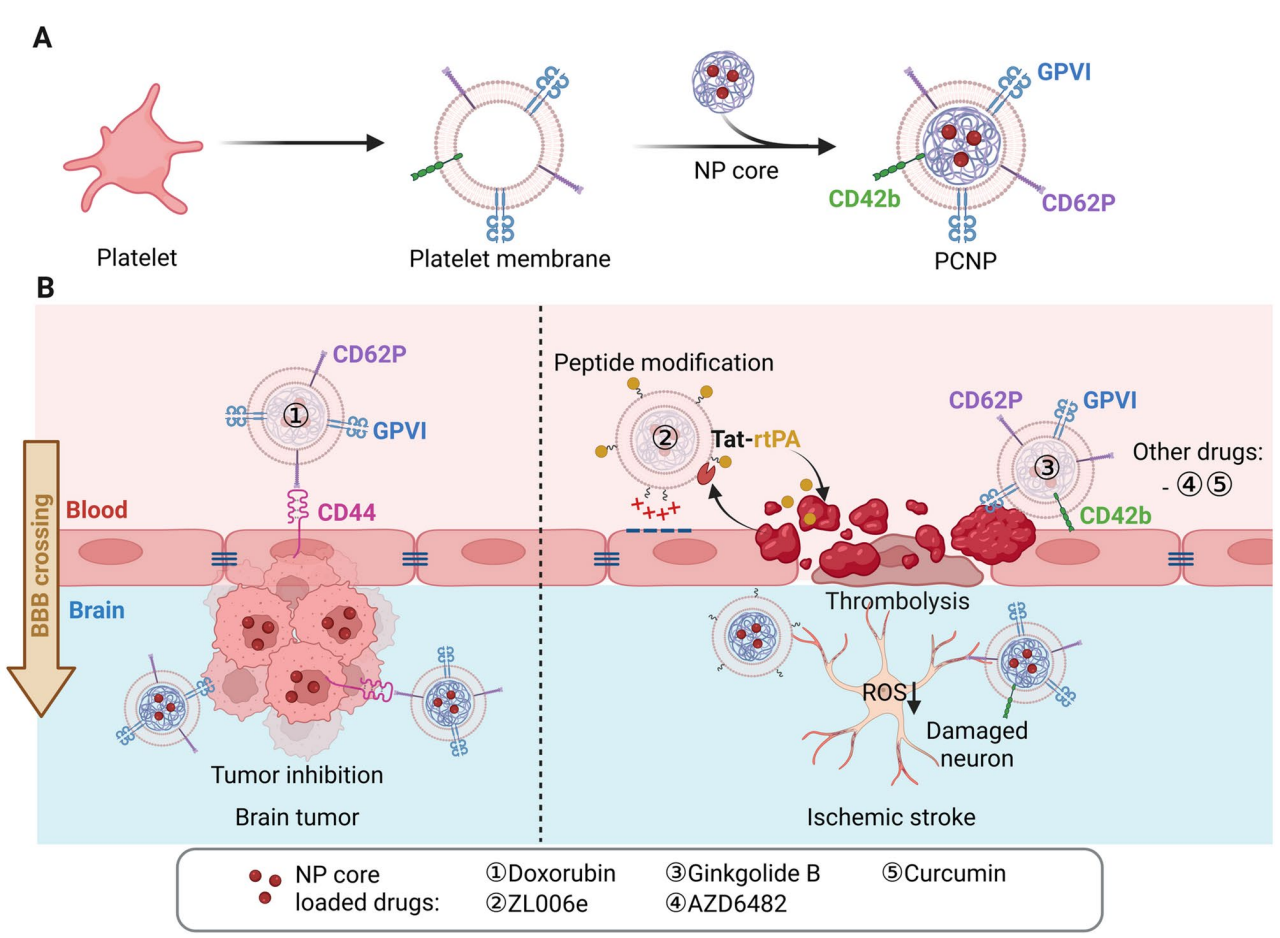
**(A)** Schematic illustration of platelet membrane-coated nanoparticle (PCNP) fabrication. **(B)** Mechanisms of PCNP-mediated BBB crossing, facilitated by platelet membrane proteins interacting with corresponding endothelial cell membrane receptors or by peptide modifications enabling BBB penetration via adsorptive transcytosis. PCNPs have been utilized for the treatment of brain tumors and ischemic stroke. The image was created using BioRender.com and is used with permission

As another anucleate cell type, platelets offer a readily accessible membrane source for nanoparticle coating. Similar to erythrocytes, platelet membranes CD47 suppress immune clearance by interacting with SIRP-α on macrophages [39]. Moreover, CD62P (P-selectin) on the platelet membranes binds selectively to CD44 on tumor cells, enabling tumor-targeting capability. Such specificity has led to the investigation of platelet membrane-engineered nanoparticles (PCNPs) as drug carriers for glioma therapy (Fig. 5) [40, 41].

Compared to free Dox and uncoated Dox-loaded nanogels (NGs), PCNG-(Dox) demonstrated superior glioma-targeting and enhanced BBB permeability, as evidenced by both in vitro transwell assays and in vivo imaging. Consequently, PCNG-treated mice exhibited significantly prolonged median survival time [40]. Although the contribution of CD62P to BBB penetration has also been considered, further mechanistic validation is required. Beyond glioma therapy, PCNPs have also been evaluated for eliminating post-surgical residual glioma cells. Thrombus formation at the damaged BBB is a major barrier to the delivery of therapeutic agents, subsequently leading to glioma recurrence. The preoperative administration of Dox-loaded PCNPs circumvented this barrier and effectively suppressed residual tumor growth, improving prognosis and extending survival in murine models [41].

The inherent roles of platelets in hemostasis, thrombosis, and inflammation enhance PCNP specificity for vascular pathologies [42, 43]. Owing to their natural affinity for injured blood vessels, platelets are key mediators in ischemic stroke progression, prompting interest in PCNPs for stroke therapy [44–49]. Cerebral arterial thrombosis, the primary cause of ischemic stroke, impedes blood flow and induces neuronal ischemia [50]. To address this, researchers developed PCNPs co-loaded with ZL006e and Tat-peptide–conjugated recombinant tissue plasminogen activator (rtPA). Following thrombus recruitment, thrombin-triggered release of rtPA promoted plasmin formation and clot dissolution. The exposed Tat peptide then facilitated BBB penetration via adsorptive transcytosis, enabling the delivery of ZL006e to ischemic neurons. This system significantly reduced infarct volume, increased ZL006e brain accumulation, and improved neurological outcomes in stroke models [45].

Beyond Tat-mediated transport, platelet surface proteins such as GPVI and CD42b contribute to BBB interaction by binding to vascular injury sites [51]. Compared to RBCNPs, PCNPs exhibited significantly enhanced BBB permeability in both in vitro and in vivo studies. Furthermore, CD62P-mediated targeting enabled ginkgolide B-loaded PCNPs to localize to ischemic and inflamed regions, mitigating inflammation and ferroptosis in stroke models [47]. Similarly, PCNP formulations carrying AZD6482 and curcumin showed improved BBB penetration and therapeutic efficacy in ischemic stroke [48, 49].

Biodistribution and safety evaluations in murine models confirmed that PCNPs do not induce observable toxicity in major organs, including the heart, liver, spleen, lungs, and kidneys [40, 46, 47]. In addition to brain accumulation, PCNPs exhibited notable uptake in the liver and kidneys, with minimal spleen retention [40, 46, 47, 49]. Reported plasma half-lives included 4.07 ± 1.05 h for tP-NP-rtPA-(ZL006e) and 2.08 ± 0.02 h for curcumin-loaded PCNPs. Although circulation times are shorter than those of RBCNPs, PCNPs display strong disease-targeting properties—particularly in ischemic stroke—highlighting their therapeutic potential.

Collectively, accumulating evidence supports the use of PCNPs as an effective brain-targeted drug delivery system for the treatment of glioma and ischemic stroke. Although platelets have a shorter lifespan than erythrocytes—resulting in reduced circulation time of PCNPs, they offer distinct advantages in disease-specific targeting. Moreover, preclinical safety evaluations have demonstrated favorable biosafety profiles, further supporting the translational potential of PCNPs for future clinical applications.

### BBB crossing mechanisms of tumor cell-derived CNPs

**Fig. 6:**
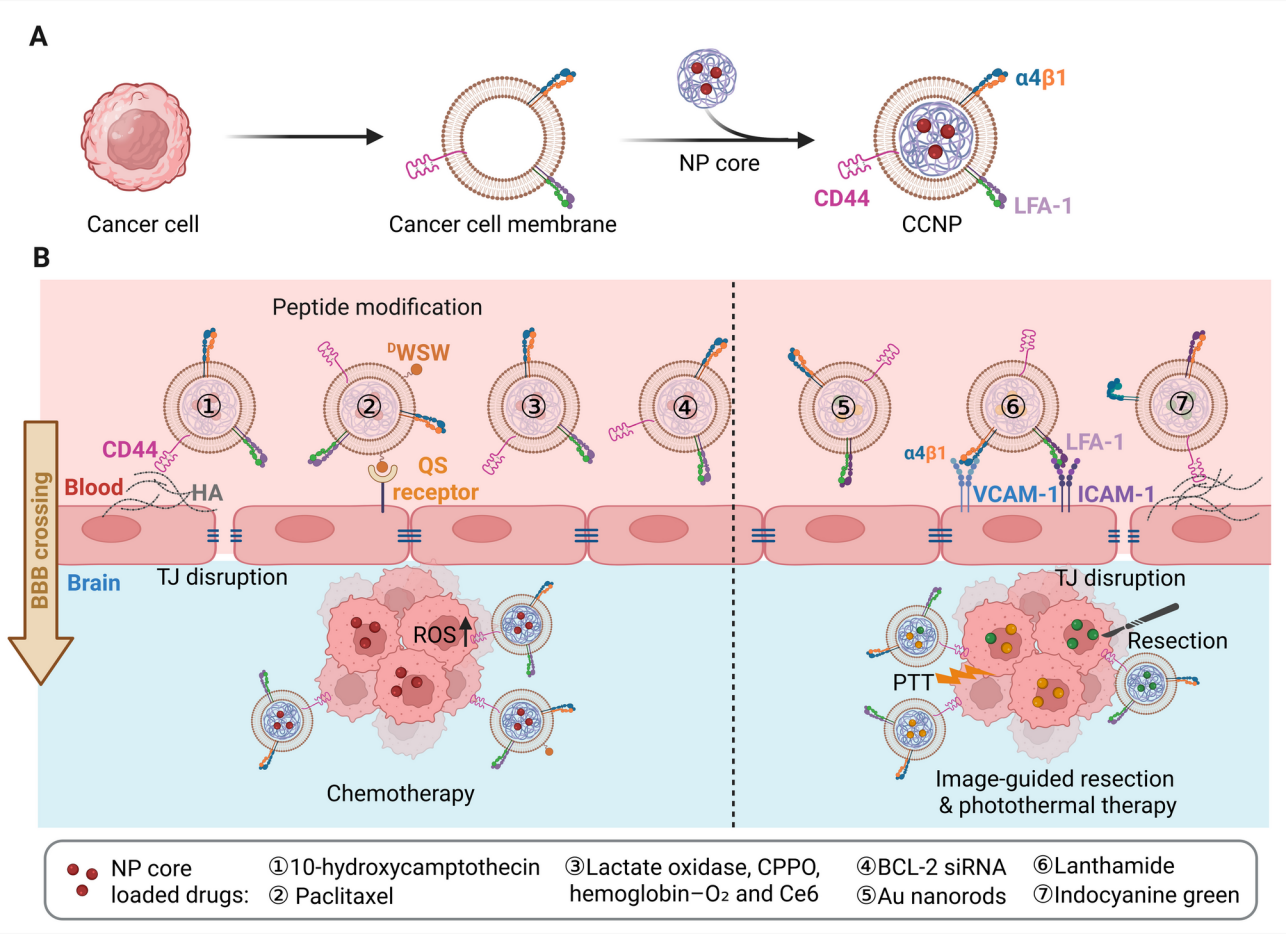
**(A)** Schematic illustration of cancer cell membrane-coated nanoparticle (CCNP) fabrication. **(B)** Mechanisms of CCNP-mediated BBB crossing, facilitated by cancer cell membrane proteins interacting with corresponding endothelial cell membrane receptors. Interactions between cell membrane proteins contribute to the disruption of tight junctions (TJs). CCNPs have been utilized for brain tumor inhibition, image-guided PTT, and image-guided tumor resection. The image was created using BioRender.com and is used with permission

Tumor cell membranes represent a widely utilized source for nanoparticle cloaking, particularly for cancer-specific drug delivery. Mechanistically, tumor cells exhibit strong adhesive properties and overexpress membrane proteins, such as CD47, CD44, and various integrins, which enable immune evasion, intercellular adhesion, and the recruitment of circulating cells—processes that contribute to tumor progression and metastasis [52]. While these features pose challenges for therapy, they have been leveraged to develop cancer cell membrane-coated nanoparticles (CCNPs), which benefit from prolonged circulation, immune escape, and enhanced tumor-targeting capabilities [19, 53]. When derived from glioma or metastatic brain tumor cells, CCNPs additionally inherit BBB-crossing and homotypic targeting properties. These characteristics have been exploited for the delivery of apoptosis-inducing agents, photothermal therapy (PTT) agents, and fluorescent probes for image-guided diagnostics and surgical resection (Fig. 6) [54–64].

CCNPs loaded with chemotherapeutic agents have demonstrated significant efficacy in glioma models. Gao et al. developed nanosuspensions (NS) cloaked with C6 glioma membranes and loaded with 10-hydroxycamptothecin (HCPT-NS/CCM) [54] or paclitaxel (CCM-(PTX)NS) [55], both of which retained key membrane markers CD47 and CD44, as confirmed via western blot. These biomimetic NSs exhibited enhanced BBB permeability in vitro and in vivo, and ^D^WSW peptide functionalization further improved drug brain accumulation. Treatment with HCPT-NS/CCM and ^D^WSW-CCM-(PTX)NS significantly reduced tumor burden and prolonged survival of glioma-bearing mice compared to uncoated or free-drug formulations [54, 55]. To exploit tumor metabolism, researchers developed a glioma-targeted system (M@HLPC) incorporating lactate oxidase (LOX), hemoglobin–O₂ (Hb–O₂), CPPO, and chlorin e6 (Ce6), cloaked with U251 glioma cell membranes. This system catalyzes lactate into pyruvic acid and H₂O₂, producing cytotoxic singlet oxygen via Ce6 and CPPO, and induces glioma cell apoptosis. Compared to uncoated controls, M@HLPCs achieved superior brain accumulation, tumor inhibition, and survival outcomes [56].

Alternatively, CCNPs have also been employed for brain-targeted siRNA delivery, which enhances reactive oxygen species (ROS)-induced tumor cell apoptosis by silencing the anti-apoptotic gene B-cell lymphoma 2 (Bcl-2). Polyethyleneimine-xanthate NPs coated with B16F10 melanoma membranes and loaded with BCL-2 siRNA (MPC@siRNA) significantly inhibited the progression of metastatic melanoma in xenograft models. Owing to the brain metastatic and BBB-penetrating properties inherited from melanoma cells, brain accumulation of MPC@siRNA was observed in tumor-bearing mice. Notably, the CCNP fabrication significantly elongated the half-life of siRNA from 0.14 to 2.4 h, which is also longer than the non-membrane controls. Interestingly, MPC@siRNA coated with B16F10 melanoma cells also showed heterogenetic targeting and inhibiting abilities in the U87MG glioma mice model, owing to the similarity of essential protein profiles between two cell lines [58].

Photothermal therapy has also been enhanced using CCNPs. Indocyanine green (ICG)-loaded polymeric nanoparticles cloaked with B16F10 or 4T1 cell membranes (PCL-ICG) achieved glioma-specific accumulation and strong tumor-selective fluorescence, with negligible signal in healthy mice. Laser irradiation of PCL-ICG-treated mice resulted in effective tumor ablation and survival extension compared to uncoated or normal cell membrane-coated controls [60]. In another study, patient-derived glioblastoma membranes were used to decorate gold nanorods (GBM-PDTCM@AuNRs), enabling dual Raman/NIR fluorescence imaging and effective PTT-guided glioma resection, leading to significantly prolonged survival [62]. Similarly, U87 glioma stem cell membrane-coated iron–nitrogen–doped mesoporous carbon nanoparticles (FNM@GSCM) enhanced BBB penetration and efficacy [63].

CCNPs have also advanced image-guided tumor resection. Lanthanide-doped nanoparticles cloaked with U87MG membranes (CC-LnNPs) facilitated high-resolution NIR-IIb imaging, enabling clear tumor–normal tissue distinction during glioma surgery. Their BBB-crossing ability was attributed to integrins LFA-1 and α4β1, which interacted with VCAM-1 and ICAM-1, transiently disrupting endothelial tight junctions [61]. Similarly, a hybrid membrane-coated ICG liposome (HM-Lipo-ICG), composed of G422 glioma and B16F10 melanoma membranes, demonstrated strong BBB permeability and glioma margin visualization under NIR guidance, enabling complete surgical resection [64].

Additionally, CCNPs can activate the immune response by serving as a source of tumor-specific antigens for immune surveillance [19]. The cancer cell membrane shell displays a repertoire of tumor-associated antigens. These antigens can be taken up by antigen-presenting cells (APCs) and processed for presentation. This process leads to the priming of tumor-specific T cells. For example, melanoma cell membrane-coated NPs loaded with the Toll-like receptor agonist (CpG) enhanced antigen presentation and multi-antigen CD8^+^ T cell responses, significantly improving tumor control in mice [65]. Similarly, an acute myeloid leukemia CCNP vaccine co-delivering leukemic antigens and an immunostimulatory adjuvant, inducing robust antigen-specific CD8^+^ T cell expansion and effector function in vivo [66]. However, evidence of CCNP-mediated immune activation in glioma models remains limited, highlighting a significant research gap in leveraging this immune mechanism for glioma-targeted therapy.

Biosafety assessments of CCNPs revealed no significant histopathological changes in major organs, as confirmed by H&E staining [54–56, 60–64, 67, 68]. Hematologic and immunologic parameters, including red and white blood cell counts, remained within normal ranges [54, 55, 64]. Liver and kidney function markers (AST, ALT, ALP, Cr, and UA) showed no abnormalities following CCNP administration [54, 58, 64]. Biodistribution studies identified the liver and spleen as primary accumulation sites, consistent across formulations [54, 55, 61, 62, 68]. Kidney uptake was also noted in specific CCNP types, suggesting formulation-dependent pharmacokinetics [56, 57, 59, 60, 67]. Despite these promising results, comprehensive pharmacokinetic characterization remains limited, warranting further investigation to support clinical translation.

In addition to the cancer cell membrane, cancer cell-secreted exosomes — a type of nanosized extracellular vesicle enriched with surface proteins — have also been utilized as coating materials for glioma therapy [69]. A recent study demonstrated that exosomes isolated from U87 glioma cells were used to coat Prussian blue nanoparticles, significantly enhancing BBB permeability and photothermal-chemotherapeutic efficacy in glioma-bearing mice [70]. In another example, polyethyleneimine-modified exosomes derived from C6 glioma cells were employed to deliver HSV-tk gene complexes, achieving superior transfection efficiency and apoptosis induction compared to non-exosomal vectors [71]. However, exosome production and isolation remain more technically challenging and less scalable than conventional membrane coating approaches. Thus, while both strategies demonstrate strong potential for CNS drug delivery, the application of CNPs is more widely utilized than exosome-coated systems.

Collectively, CCNPs derived from homotypic tumor cells have demonstrated promising BBB penetration and tumor-targeting capabilities. When combined with fluorescent agents, these nanoparticles have been successfully employed in advanced PTT and surgical resection, offering potential for the development of innovative strategies for clinical cancer diagnosis and treatment. However, obtaining sufficient homotypic tumor cells from patients remains a challenge. Once acquired, efficient and scalable CNP fabrication is essential to support personalized therapeutic approaches. At present, most CNP membranes are sourced from laboratory-cultured stable cell lines, while the use of primary patient-derived cells for CNP fabrication remains in its early stages. Further optimization and technological development are necessary to advance this strategy toward clinical applications.

### BBB crossing mechanisms of Leukocyte-derived CNPs

Unlike erythrocytes and platelets, cloaking nanoparticles with leukocyte-derived membranes is more complex due to the presence of nuclei and intracellular organelles. However, leukocytes play critical roles in inflammatory responses, endowing leukocyte membrane-coated nanoparticles with unique advantages for targeted drug delivery in inflammatory and pathological conditions [16].

**Macrophages**—key phagocytic cells of the innate immune system—express a wide array of surface receptors that mediate biological signal recognition and confer innate homing ability to inflamed and tumor tissues. Accordingly, macrophage membrane-coated nanoparticles (MCNPs) have emerged as a promising biomimetic platform for the diagnosis and treatment of tumors, inflammatory diseases, and infections [72].

**Fig. 7:**
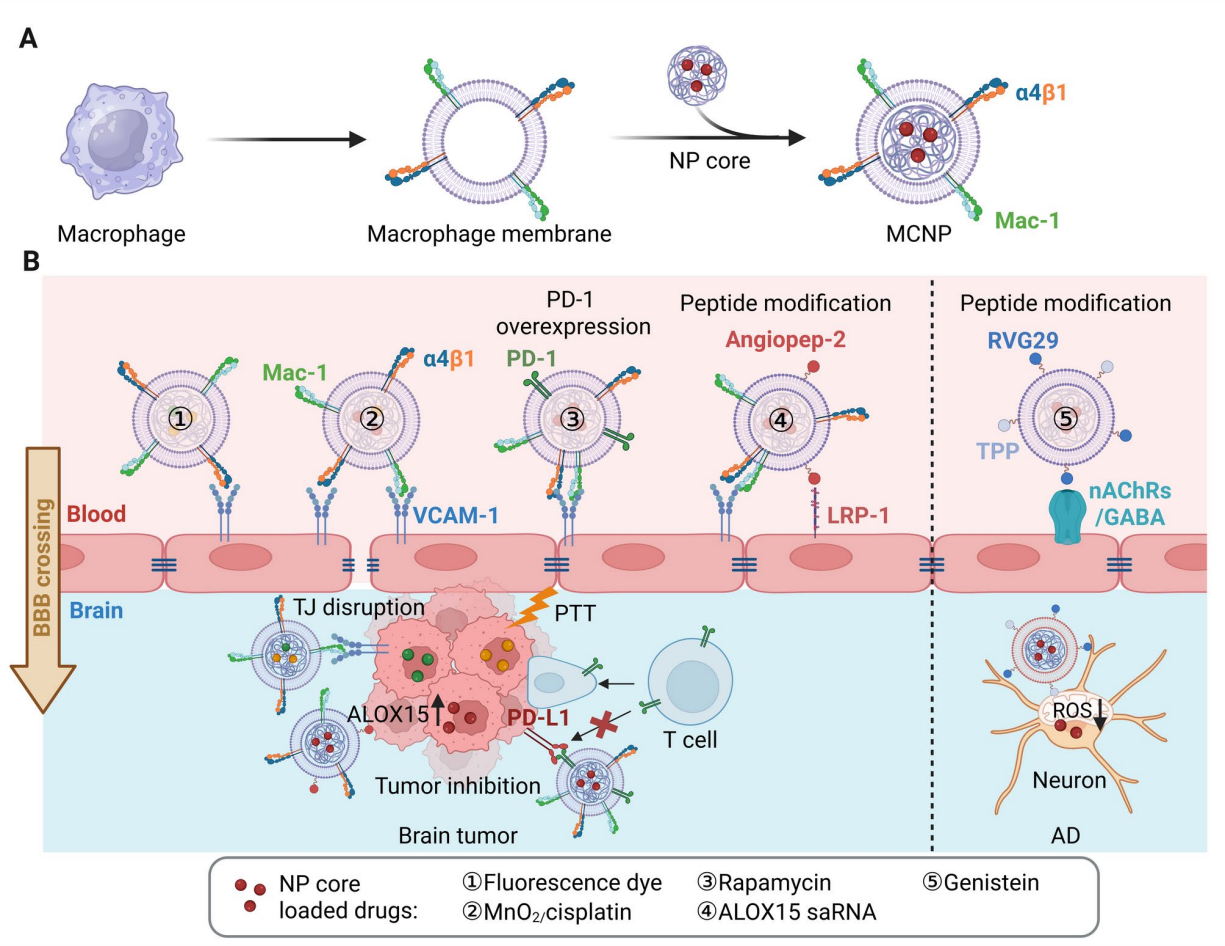
**(A)** Schematic illustration of macrophage membrane-coated nanoparticle (MCNP) fabrication. **(B)** Mechanisms of MCNP-mediated BBB crossing, facilitated by macrophage membrane proteins or peptide modifications interacting with corresponding endothelial cell membrane receptors, lead to the disruption of tight junctions (TJs). MCNPs have been utilized for the treatment of brain tumors and Alzheimer’s disease (AD). The image was created using BioRender.com and is used with permission

MCNPs have demonstrated potential in glioma-targeted drug delivery [21]. Surface proteins such as integrin α4, β1, and macrophage-1 antigen (Mac-1; integrin αmβ2) facilitate BBB penetration by interacting with vascular cell adhesion molecule-1 (VCAM-1) on endothelial cells, promoting tight junction disruption (Fig. 7) [73]. Although further validation is warranted, MCNPs have been shown to modulate tight junction proteins such as ZO-1, facilitating BBB transport [74]. Additionally, VCAM-1 is overexpressed on glioma cells, enhancing MCNP binding and tumor-targeting capabilities. Glioma-associated upregulation of VCAM-1 in endothelial cells may further increase MCNP permeability under pathological conditions [74, 75]. Notably, glioma cells have also been implicated in the upregulation of VCAM-1 on endothelial cells, further increasing MCNP permeability under pathological conditions [76]. Based on these interactions, multiple MCNP-based strategies have been investigated for glioma drug delivery [22, 74, 75, 77–79].

MCNPs have also enabled PTT. For example, macrophage membrane-cloaked nanoparticles loaded with IR-792 (MDINPs) achieved targeted tumor accumulation and photothermal ablation in glioma-bearing mice. Compared to free IR-792 and uncoated IR-792-loaded nanoparticles, MDINPs demonstrated enhanced BBB permeability, reduced tumor volume, and prolonged survival following NIR irradiation [74]. Alternatively, manganese dioxide (MnO₂) and cisplatin co-loaded nanogels (MPM@PNG), coated with macrophage membranes, enabled chemodynamic therapy under MR imaging guidance. MPM@PNG exhibited superior brain accumulation and anti-tumor efficacy compared to free cisplatin, MnO₂-loaded NPs, and uncoated nanogels [75]. Similarly, MCNPs co-loaded with photosensitizer Ce6 and chemotherapeutic drug temozolomide (TMZ) efficiently traversed the BBB in the mouse model. Upon precise intracranial laser exposure, Ce6 activates photodynamic therapy (PDT), which works together with TMZ’s chemotherapeutic effects, resulting in a combined therapy that effectively eradicates glioma cells [77].

BBB transport and glioma targeting of MCNPs can be further enhanced by genetic engineering or ligand modification. For instance, nanoparticles cloaked with membranes from PD-1-overexpressing macrophages blocked PD-L1 on glioma cells, reactivating CD8⁺ cytotoxic T cells and attenuating tumor immune evasion [22]. Additionally, Angiopep-2 modified MCNPs loaded with ALOX15 small activating RNA (saRNA) were developed to induce ferroptosis in glioblastoma. This system (Ang-MCsaNP) significantly improved BBB penetration and tumor accumulation in glioma-bearing mice relative to uncoated nanoparticles [78].

Beyond glioma, MCNPs have been investigated for neurodegenerative and neurological disorders. In AD models, MCNPs functionalized with RVG29 and triphenylphosphonium (TPP) delivered Genistin to neuronal mitochondria, reducing oxidative stress and alleviating AD symptoms [80]. In epilepsy models, macrophage membrane-coated nanoparticles carrying TC-DAPK6 penetrated the BBB and targeted inflamed brain regions, providing neuroprotective effects [81].

Biosafety assessments in murine models revealed no significant morphological or pathological abnormalities in the heart, liver, spleen, lungs, kidneys, or brain following MCNP administration [22, 74, 75, 77, 80]. The plasma half-life of MCNPs (~ 3 h) exceeds that of uncoated nanoparticles but remains shorter than RBCNPs [75, 77]. Biodistribution patterns varied by formulation: MPM@PNG and PD-1-MM@RAPA exhibited spleen accumulation with minimal kidney presence [22, 75], while Ce6-TMZ-loaded MCNPs preferentially accumulated in the kidneys, with negligible spleen distribution [77]. Liver accumulation was consistently observed across all MCNP types, identifying it as the primary metabolic organ.

**Fig. 8:**
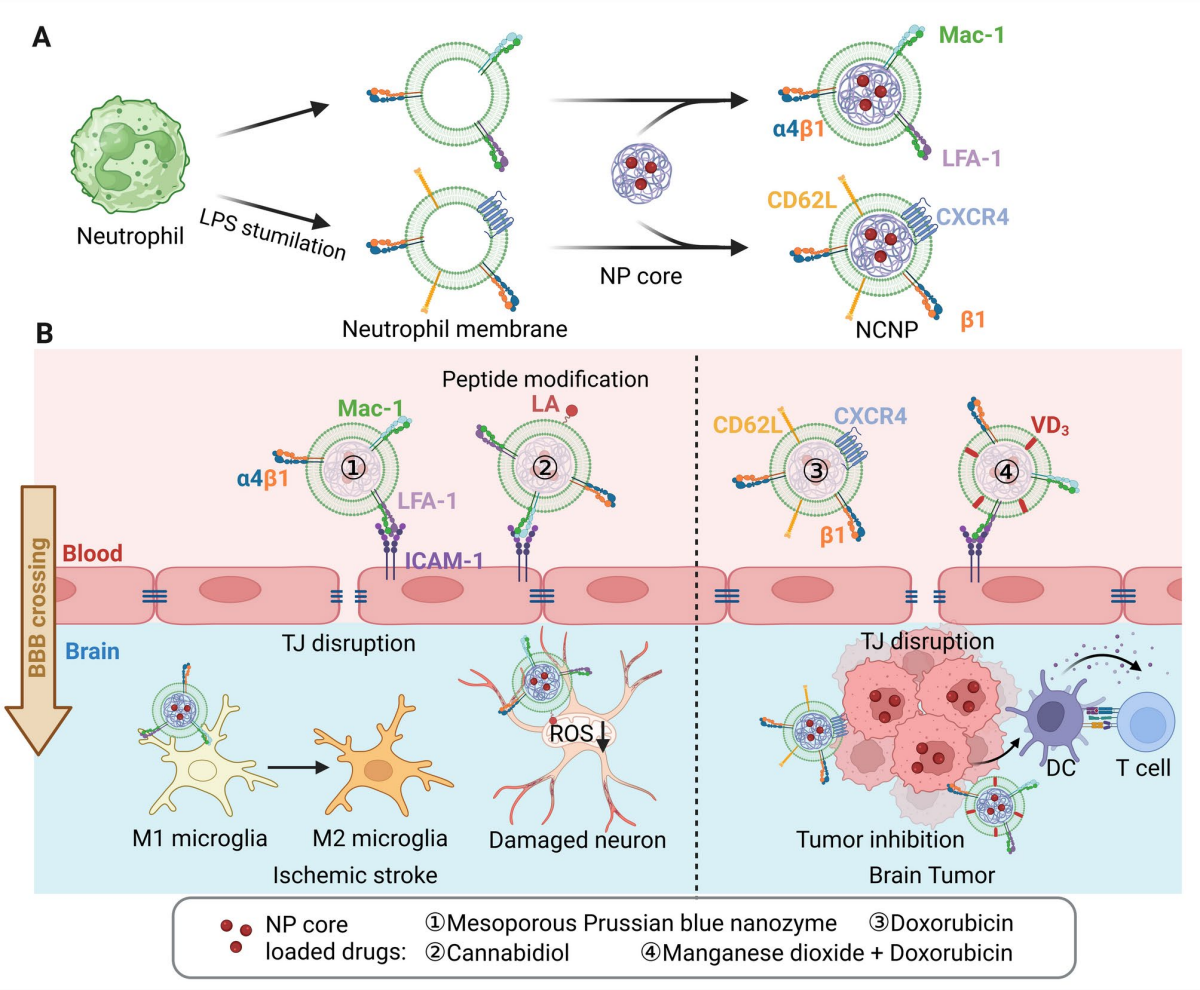
**(A)** Schematic illustration of neutrophil membrane-coated nanoparticle (NCNP) fabrication. **(B)** Mechanisms of NCNP-mediated BBB crossing, facilitated by neutrophil membrane proteins interacting with corresponding endothelial cell membrane receptors, lead to the disruption of tight junctions (TJs). NCNPs have been utilized for the treatment of ischemic stroke and brain tumors. The image was created using BioRender.com and is used with permission

**Neutrophils** are rapidly recruited to ischemic sites in response to pro-inflammatory cytokines released from injured brain tissue, a phenomenon observed in both murine models and human stroke specimens [82, 83]. Similar to macrophages, neutrophil surface proteins—including lymphocyte function-associated antigen 1 (LFA-1), integrin α4β1, and Mac-1—interact with intercellular adhesion molecule-1 (ICAM-1) on brain endothelial cells to facilitate BBB penetration (Fig. 8) [82]. Leveraging this natural targeting ability, neutrophil membrane-coated nanoparticles (NCNPs) have been explored for drug delivery in ischemic stroke and glioma therapy.

One such platform, MPBzyme@NCM—a mesoporous Prussian blue nanozyme cloaked in neutrophil membrane—was developed to alleviate oxidative stress, suppress neuroinflammation, and improve neurological outcomes after ischemic stroke. The anti-inflammatory effect was mediated by competitive ICAM-1 binding, which reduced neutrophil infiltration and promoted microglial polarization from the pro-inflammatory M1 to the anti-inflammatory M2 phenotype. In ischemic mice, neutrophil membrane coating significantly enhanced brain accumulation and resulted in improved therapeutic efficacy [84]. To address mitochondrial dysfunction and reactive oxygen species (ROS) overproduction following acute ischemia, α-lipoic acid (LA)-decorated NCNPs loaded with cannabidiol (CBD) (LA-MN-NP/CBD) were engineered for neuroprotection. These formulations demonstrated rapid and enhanced brain accumulation and significantly reduced infarct volume while improving neurological function in ischemic mice compared to uncoated nanoparticles [85].

Neutrophil-driven inflammation has also been implicated in glioma progression, promoting their recruitment across the BBB [86]. Capitalizing on this, NCNPs have been developed for glioma-targeted therapy. A Dox-loaded NCNP formulation was tested in both bEnd.3 transwell assays and glioma-bearing nude mice, showing enhanced brain accumulation and post-surgical tumor suppression. Lipopolysaccharide (LPS) activation of neutrophils increased the expression of integrin β1, CXCR4, and CD62L, which were retained on NCNPs and correlated with improved BBB permeability and glioma targeting. Mice treated with LPS-activated NCNPs exhibited the longest median survival (37 days) compared to non-activated NCNPs (25 days) and uncoated nanoparticles (27 days) [87]. Similarly, neutrophil membrane-coated MnO₂ nanoparticles exhibited enhanced BBB transport and glioma suppression. Membrane proteins such as integrin α4β1 and CD18 facilitated BBB penetration via tight junction modulation and mediated tumor adhesion. To further improve immune activation and therapeutic efficacy, researchers fused neutrophil membranes with HSPC liposomes incorporating vitamin D_3_, enabling cGAMP delivery and dendritic cell (DC) activation via the cGAS–STING pathway. This resulted in enhanced CD8⁺ T cell infiltration into the tumor microenvironment [88].

Biodistribution studies revealed predominant NCNP accumulation in the liver and spleen [84, 88], with moderate kidney uptake observed in some formulations [85, 87]. Histological analysis showed no significant tissue damage in major organs following NCNP administration [84, 85, 87, 88]. However, comprehensive pharmacokinetic data remain limited and must be addressed to support clinical translation.

**Natural killer (NK) cells** exhibit a distinct anti-tumor mechanism that does not rely on antigen presentation but is instead triggered by the recognition of stress-induced ligands overexpressed on malignant cells. This interaction activates NK cells and initiates targeted cytolysis [89]. Similar to other leukocytes, NK cells can disrupt blood-brain barrier (BBB) integrity by interacting with adhesion molecules such as VCAM-1 and ICAM-1 on brain endothelial cells. Deng et al. demonstrated that NK cell membrane-coated nanoparticles (NKCNPs) retained this BBB-crossing ability, mediated by integrin α4β1 and LFA-1 binding to VCAM-1 and ICAM-1, respectively, resulting in downregulation of tight junction proteins such as ZO-1 [90].

These NKCNPs, incorporating near-infrared II (NIR-II) aggregation-induced emission (AIE)-active conjugated polymers, enabled both high-resolution NIR-II fluorescence imaging and image-guided photothermal therapy in glioma-bearing mice, significantly suppressing tumor growth [90]. Functionalization with the cRGD peptide further enhanced BBB penetration and glioma targeting through interaction with integrin αvβ3 expressed on brain endothelial and glioblastoma cells. When loaded with TMZ and interleukin-15 (IL-15), cRGD-modified NKCNPs promoted dendritic cell maturation and NK cell activation, leading to significant tumor reduction and extended survival in glioma models [91].

Importantly, NK cell membrane coating increased nanoparticle circulation time, extending the elimination half-life from 5.6 h (uncoated) to 8.5 h. cRGD-modified NKCNPs exhibited an even longer half-life (t₁/₂ = 10.69 h), markedly exceeding that of free TMZ (t₁/₂ = 1.99 h), highlighting the pharmacokinetic advantages conferred by NK membrane cloaking and ligand functionalization.

Collectively, the critical role of leukocytes in inflammatory responses endows leukocyte membrane-camouflaged nanoparticles with unique advantages for targeted drug delivery in inflammatory conditions, including brain tumors and ischemic stroke. The presence of specific membrane proteins, inherited by CNPs, enables BBB penetration through tight junction modulation in a biologically natural and non-disruptive manner, thereby minimizing the risk of infection. Moreover, peptide functionalization and advanced genetic engineering have further enhanced the BBB permeability and disease-targeting capabilities of leukocyte-derived CNPs. However, pharmacokinetic data for NCNPs and comprehensive biodistribution profiles for NKCNPs remain limited, highlighting the need for further investigation to support clinical translation.

### BBB crossing mechanisms of hybrid CNPs

Although the BBB permeability and disease-targeting capabilities of CNPs with various membrane cloaking have been well demonstrated, single-cell membrane coatings present notable limitations. For instance, while erythrocyte- and platelet-derived CNPs offer prolonged circulation times, their tumor-targeting efficiency remains suboptimal without additional surface modifications. To overcome these challenges, hybrid membrane-coated nanoparticles have been developed to integrate the complementary advantages of multiple cell types [92].

For example, β-Mangostin-loaded NPs cloaked with a platelet-C6 glioma hybrid membrane exhibited both enhanced immune escape and improved glioma-targeting ability compared to C6 glioma CNPs or platelet CNPs. Following hybridization, platelet-C6-CNPs show increased brain accumulation and tumor-targeting properties compared to C6 glioma or platelet-CNPs alone. Consequently, glioma-bearing mice injected with platelet-C6 hybrid CNPs exhibited the smallest tumor sizes and the highest survival rates among all experimental groups treated with different NPs [93]. Similarly, a hybrid membrane composed of erythrocyte and brain-metastatic 231Br tumor cells improved nanoparticle circulation, BBB penetration, and tumor suppression when loaded with dexamethasone and embelin. Remarkably, the circulation half-life of hybrid CNPs (16.820 ± 1.778 h) was similar to that of RBCNP (17.194 ± 4.396) [94]. In another study, a triple-membrane hybrid composed of erythrocytes, U251 glioma cells, and macrophages integrated key membrane markers from each source—CD47 for immune evasion, CD44 for glioma targeting and BBB transport, and CD86 for immune modulation. These multifunctional CNPs exhibited superior systemic circulation, immune escape, BBB permeability, and glioma-specific accumulation in murine models [95].

Although membrane hybridization represents a promising strategy to overcome the current limitations of CNPs, research on hybrid membrane-coated CNPs for BBB penetration remains in its early stages. Comprehensive data on physicochemical characterization, biodistribution, and biosafety are still limited, indicating that substantial investigation is required before clinical translation can be realized.

In summary, CNPs employ a diverse array of mechanisms to traverse the BBB, depending on their membrane origin and surface modifications. These mechanistic insights provide a foundation for the rational design of CNPs to overcome the BBB and achieve targeted delivery for CNS diseases (Table 2).

**Table 2:** Cell membrane-engineered nanomedicine for CNS diseases

Types of CNP	Modification	Membrane protein	Target	Loaded drug	Disease	T_1/2_ (h)	In vivo brain distribution	Clinical state	References
RBCNPs	^D^CDX peptide	-	nAChRs	Doxorubin	Glioma	33.3±4.1	0.3ug per gram of organ (14days)	Preclinical	[30]
	c(RGDyK)	-	Integrin αvβ3	Docetaxel	Glioma	19.7±1.1	20ug per gram of organ (24 h)	Preclinical	[32]
	Angiopep-2	-	LRP-1	Doxorubin + Lexiscan	Glioma	9.3	9.66% of injected dose per gram of tissue (8 h)	Preclinical	[31]
	^D^WSW/NGR	-	QS receptor/CD13	Euphorbia factor L1	Glioma	-	Significantly increased brain accumulation	Preclinical	[33]
	RVG29/TPP	-	nAChRs or GABA	Resveratrol	AD	8.06±0.39	Brain accumulation identified 1 h post-injection	Preclinical	[34]
	TfR-A	-	TfR	CD22 shRNA	AD	-	Significantly increased brain accumulation	Preclinical	[35]
PCNPs	-	CD62P	CD44	Doxorubin	Glioma	-	Significantly increased brain accumulation	Preclinical	[40, 41]
	Tat-rtPA	-	-	ZL006e	Ischemic stroke	4.07±1.05	Significant brain accumulation identified 2 h post-injection	Preclinical	[45]
	-	GPVI/CD42P	Vascular injury sites	ginkgolide B	Ischemic stroke	-	-	Preclinical	[47]
	-	GPVI/CD42P	Vascular injury sites	AZD6482	Ischemic stroke	-	-	Preclinical	[48]
	-	GPVI/CD42P	Vascular injury sites	Curcumin	Intracerebral hermorrhage	2.083±0.0021	Significantly increased brain accumulation	Preclinical	[49]
CCNPs	-	CD44	HA	10-hydroxycamptothecin	Glioma	-	Increased brain accumulation identified 2 h post-injection	Preclinical	[54]
	^D^WSW	-	QS receptor	Paclitaxel	Glioma	-	Increased brain accumulation identified 2 h post-injection	Preclinical	[55]
	-	-	-	Lox, Hb-O_2_, CPPO and Ce6	Glioma	-	Significantly increased brain accumulation	Preclinical	[56]
	-	-	-	Temozolomide + cisplatin	Glioma	7.84	Significantly increased brain accumulation	Preclinical	[57]
	-	-	-	BCL-2 siRNA	Glioma	2.4	Increased brain accumulation identified 2 h post-injection	Preclinical	[58]
	-	-	-	Paclitaxel + PGK1 siRNA	Glioma	3.5	Significantly increased brain accumulation	Preclinical	[59]
	-	-	Cell surface receptors	Indocyanine green	Glioma	12.24	Increased brain accumulation identified 4 h post-injection	Preclinical	[60]
	-	-	Cell surface receptors	Au nanorods	Glioma	-	Increased brain accumulation	Preclinical	[62]
	-	-	-	Doxorubicin loaded Fe-N doped nanospheres	Glioma	-	Increased brain accumulation identified 2 h post-injection	Preclinical	[63]
	-	Integrin α4β1, CD44, LFA-1	HA, ICAM-1, VCAM-1	Lanthamide	Glioma	-	Increased brain accumulation identified 1 h post-injection	Preclinical	[61]
	-	CD44	HA	Indocyanine green	Glioma	-	Significant brain accumulation identified 6 h post-injection	Preclinical	[64]
MCNP	-	Integrin α4β1, Mac-1	VCAM-1	IR-792	Glioma	-	Significant brain accumulation identified 12 h post-injection	Preclinical	[74]
	-	Integrin α4β1, Mac-1	VCAM-1	MnO_2_ + cisplatin	Glioma	3.37	Increased brain accumulation identified 1 h post-injection	Preclinical	[75]
	-	Integrin α4β1, Mac-1	VCAM-1	Ce6 + temozolomide	Glioma	3.011	Increased brain accumulation	Preclinical	[77]
	PD-1 overexpression	Integrin α4β1, Mac-1	VCAM-1	Rapamycin	Glioma	-	Significantly increased brain accumulation	Preclinical	[22]
	Angiopep-2	Integrin α4β1, Mac-1	LRP-1, VCAM-1	ALOX15 saRNA	Glioma	9.20±0.21	Increased brain accumulation identified 1 h post-injection	Preclinical	[78]
	RVG29/TPP	-	nAChRs or GABA	Genistein	AD	-	Significantly increased brain accumulation	Preclinical	[80]
NCNP	-	Integrin α4β1, Mac-1, LFA-1	ICAM-1	Mesoporous Prussian Blue Nanozyme	Ischemic stroke	-	Increased brain accumulation	Preclinical	[84]
	α-lipoic acid	Integrin α4β1, Mac-1, LFA-1	ICAM-1/mitochondria	Cannabidiol	Ischemic stroke	-	Increased brain accumulation identified 2 h post-injection	Preclinical	[85]
	-	Integrin β1, CD62L, CXCR4	-	Doxorubicin	Glioma	-	Significantly increased brain accumulation	Preclinical	[87]
	Vitamin D_3_ Insertion	Integrin α4β1, Mac-1, LFA-1	ICAM-1	MnO_2_ + Doxorubicin	Glioma	-	Increased brain accumulation identified 2 h post-injection	Preclinical	[88]
NKCNP	-	Integrin α4β1, LFA-1	VCAM-1, ICAM-1	NIR-II AIE polymer	Glioma	8.5	Increased brain accumulation identified 6 h post-injection	Preclinical	[90]
	cRGD	Integrin α4β1, LFA-1	VCAM-1, ICAM-1, Integrin αvβ3	Temozolomide + IL-15	Glioma	10.69	Increased brain accumulation	Preclinical	[91]
Hybrid CNPs	Platelet + glioma cell	-	-	β-Mangostin	Glioma	6.16	Significantly increased brain accumulation compared to mono-coated CNPs	Preclinical	[93]
	Erythrocyte + brain metastatic cell	ALCAM, CD44, ICAM-1 LFA-1 GD1α	-	Dexamethasone + embelin	Brain metastases	16.820±1.778	Significantly increased brain accumulation compared to mono-coated CNPs	Preclinical	[94]
	Erythrocyte + macrophage + glioma cell	CD47, CD86, CD44	-	Lomitapide loaded terrahedral DNA nanocage	Glioma	-	Increased brain accumulation	Preclinical	[95]

## Future perspectives

With the rising prevalence of CNS disorders, brain-targeted diagnostic and therapeutic agents have garnered increasing attention, where BBB permeability is a critical factor in drug delivery technologies. Among the existing nanocarriers, CNPs camouflaged with natural membranes derived from erythrocytes, platelets, tumor cells, and leukocytes have demonstrated significant BBB penetration and disease-specific targeting abilities, offering promising avenues for the treatment of brain tumors, ischemic stroke, and AD. While significant progress has been made in preclinical models, the leap from experimental success to clinical application demands both mechanistic refinement and scalable, Good Manufacturing Practice (GMP)-compliant manufacturing processes.

### Challenges of CNP clinical translation

Due to their accessibility and low cost, preclinical studies have predominantly employed blood cells from animal sources or human cancer cell lines cultured in vitro to fabricate BBB-crossing CNPs. However, CNPs derived from these sources are prone to rapid clearance by the host immune system, thereby compromising delivery efficacy and eliciting adverse immune reactions [96]. In contrast, membranes obtained from primary human cells offer improved immunocompatibility, but are limited by challenges in cell sourcing, human leukocyte antigen matching, and ethical constraints, making them impractical for large-scale manufacturing [97, 98]. To overcome these translational bottlenecks, genetically engineered cells represent a promising alternative for the scalable manufacture of CNPs. Genome editing strategies—such as the knockout of immunogenic epitopes (e.g., α-Gal) or the introduction of human-specific “self-markers” like CD47—can significantly reduce immune recognition and prolong systemic circulation [99, 100]. In parallel, advances in the large-scale expansion of primary human cells under clinically compliant conditions may further facilitate the clinical translation of CNP-based therapeutics in the future.

The industrial-scale production of CNPs remains a major unresolved challenge, representing a critical barrier for clinical translation [101]. Current fabrication methods primarily include physical extrusion, sonication, and microfluidics. Physical extrusion effectively preserves membrane integrity and functionality through mechanical compression that avoids lipid perturbation, but the process is labor-intensive and time-consuming. Sonication is a rapid and straightforward alternative; however, it may damage membrane structure through bilayer destabilization due to cavitation forces, leading to compromised membrane and protein functionality. Microfluidic approaches offer better control over assembly but typically exhibit limited throughput and require precise control of multiple fabrication parameters [19]. To date, these methods remain confined to the laboratory scale and exhibit batch-to-batch variability. Among them, microfluidics holds promise for industrial scale-up, particularly with the emergence of high-throughput, continuous-flow microfluidic systems capable of producing uniformly coated nanoparticles, as recently demonstrated in applications for cancer diagnostics [97, 102]. Furthermore, preserving the bioactivity of membrane proteins during long-term storage and transportation remains a persistent challenge due to their environmental sensitivity [27], underscoring the need for improved preservation strategies.

In addition to the need for standardized bulk-scale synthetic methodologies, the clinical translation of CNPs will require rigorous quality control. Vora et al. emphasized the importance of standardized characterization assays to assess membrane integrity, protein orientation, and functional stability [103]. Integration of real-time analytical methods such as microfluidic-assisted flow cytometry or nanoparticle tracking analysis (NTA) could enable continuous monitoring of coating consistency, paving the way for adaptive process optimization. In addition to these functional parameters, ensuring the efficacy of CNPs will require monitoring across numerous physical and chemical properties that are only beginning to be understood, and which may vary considerably with variation in the donor cells—especially in the case of patient-derived cell membranes. Membrane fluidity, in particular, has been shown to play an important role in CNP performance. The addition of fluidity-enhancing supplemental phospholipids greatly increased the production yield of intact membranes in CNPs cloaked by colon carcinoma-derived membranes [104], while cholesterol reduction in T-cell membrane-coated CNPs led to optimized immune evasion [105]. The rigidity of the nanoparticle core has also been shown to strongly influence the properties of mesenchymal stem cell-derived CNPs, with softer particles having reduced immune cell uptake and a higher degree of cancer targeting [106]. Heterogeneous lipid rafts with reduced fluidity are well known to control a wide range of cellular and protein functions, making it unsurprising that similar effects have been observed in CNPs [107, 108]. However, the extent to which these observed effects are related to parent cell raft content remains unexplored. For the development of Current Good Manufacturing Practice compliant production processes, a greater understanding of these properties, optimization of fabrication parameters, and integration of real-time monitoring systems will be essential.

While natural cell membrane cloaking has significantly improved drug delivery across the BBB, additional modification, such as the insertion of targeting peptides or expression of functional proteins, is often necessary to achieve optimal BBB penetration and disease specificity, as previously described. However, conventional chemical conjugation methods are typically time-consuming and prone to batch-to-batch variability. To overcome these limitations, Zhang et al. recently developed a novel genetic engineering-based approach for modular modification of cell membranes, enabling efficient CNP development for broader therapeutic applications. This approach involves the establishment of a stable membrane SpyCatcher-expressing cell line, which enables precise and reproducible conjugation with tumor-specific ligands labeled with SpyTag. Leveraging the high-affinity SpyCatcher–SpyTag interaction, targeting moieties can be anchored to the membrane surface in a scalable manner, facilitating the rapid fabrication of ligand-functionalized CNPs. This platform was successfully validated in an ovarian cancer model, where the ligand-functionalized SpyCatcher–CNPs with Dox loading exhibited enhanced tumor targeting and potent antitumor efficacy both in vitro and in vivo [109]. Looking forward, this modular engineering strategy holds strong potential for adaptation to BBB-targeted drug delivery. Moreover, its reproducibility and scalability make it an attractive solution for industrial-scale production under standardized manufacturing protocols.

The use of patient-derived cells for the personalized fabrication of CNPs holds significant promise, aligning with the trend of development toward individualized therapeutics. In particular, CNPs engineered from autologous blood or tumor cells offer considerable potential for tailored brain-targeted drug delivery across the BBB. However, this approach remains in its early stages. Existing studies are predominantly based on short-term murine models and lack comprehensive assessments of long-term safety, biodistribution, and pharmacokinetics [20]. Thus, more robust in vivo investigations are urgently needed to establish the long-term toxicological and pharmacological profiles of human-derived CNPs. From a manufacturing perspective, the scalable and GMP-compliant acquisition of patient-specific membrane materials remains a critical technical barrier. Achieving a rapid, continuous, and standardized supply of clinically relevant human membranes presents considerable logistical challenges. In parallel, the ethical and regulatory oversight of CNPs, due to their hybrid nature as both biologically derived and nanoscale entities, does not neatly align with existing nanomedicine regulatory frameworks. This underscores the need for dedicated guidelines and ethical review mechanisms tailored to the unique properties of CNPs.

To date, there is no therapeutic formulation utilizing cell membrane biomimetic-coated nanoparticles that has received formal approval for clinical use by either the FDA or the European Medicines Agency (EMA) [110]. However, the therapeutic agents encapsulated within CNP cores, such as Dox, are already approved in conventional formulations, including solutions and liposome [10]. Therefore, the key hurdles for regulatory approval are mainly related to the coating of biomimetic materials. Moving forward, collaborative efforts among academic researchers, clinical institutions, industry stakeholders, and regulatory bodies will be essential to enable the safe and effective clinical translation of personalized CNP platforms.

### Artificial intelligence in contributing to CNP clinical translation

The design and characterization of BBB-crossing CNPs for clinical application in CNS diseases involves the monitoring of an expansive array of parameters, encompassing nanoparticle composition, membrane source selection, fabrication optimization, and surface functionalization with peptides or proteins. Optimizing such multifactorial systems is a complex and data-intensive challenge. Fortunately, recent advances in artificial intelligence (AI) and machine learning offer powerful in-silico tools to accelerate and refine the process of biomedical discovery [111]. Although AI has been employed to predict the BBB permeability of nanoparticle-based drug delivery systems and optimize their design for enhanced brain-targeting efficiency, its integration into CNP development remains limited but highly promising. By integrating data on functional peptides, surface proteins, and biotoxicity, deep learning models may reveal critical factors governing CNP behavior in vivo, thereby facilitating and accelerating clinical translation.

AI-based models were utilized to predict peptides facilitating BBB-penetration. Augur is a machine learning-based framework established to accurately predict BBB-penetrating peptides for nanoparticle functionality. By integrating multiple sequence-derived physicochemical features and applying a data augmentation strategy to address sample imbalance, the model achieved superior performance (AUC > 0.93) on both training and independent test sets, outperforming existing tools [112]. Building on this success, similar modeling approaches could be extended to CNPs. Such tools can streamline high-throughput screening, optimize feasible membrane proteins or functional peptides, and enable the de novo design of CNPs with improved BBB permeability and disease-targeting specificity. These advances hold particular relevance for engineering CNPs with membrane-displayed ligands or fusion proteins to facilitate BBB traversal.

In parallel, machine learning algorithms have shown increasing utility in nanotoxicology, enabling the prediction of adverse effects based on physicochemical descriptors [113]. A landmark study by Leszczynski et al. in 2011 pioneered the use of nano-QSAR (quantitative structure–activity relationship) modeling to predict the cytotoxicity of metal oxide nanoparticles, laying the foundation for AI-driven safety assessments [114]. Since then, nano-QSAR models have been refined to assess toxicity and biological behavior across various nanoparticle types [115, 116]. Extending these approaches to CNPs, future frameworks that integrate high-quality datasets with deep generative models and graph-based neural networks could enable simultaneous optimization of CNP efficacy and safety, supporting a safe-by-design strategy in precision nanomedicine.

Recent breakthroughs in AI-enhanced biodistribution analysis have provided unprecedented resolution and scalability in characterizing nanoparticle behavior in vivo. For instance, Huang et al. developed a U-Net-based deep learning segmentation method that enabled single-vessel quantification of nanoparticle permeability across diverse tumor vasculatures. By analyzing over 67,000 tumor blood vessels, the study distinguished between passive and active transport mechanisms and guided the rational engineering of protein nanocarriers for enhanced performance in low-permeability tumors [117]. Complementarily, Erturk et al. introduced SCP-Nano, a system combining tissue clearing, 3D deep learning, and advanced microscopy to map nanocarrier distribution across the entire mouse body at single-cell resolution. This platform accurately tracked multiple nanocarrier types and revealed off-target accumulations linked to molecular changes in tissues [118]. In the future, combining AI-driven imaging and design pipelines with CNPs, with the integration of membrane-specific signatures, could enable specific and comprehensive development by closing the loop from biodistribution mapping to targeting of mechanism-specific transport.

**Fig. 9:**
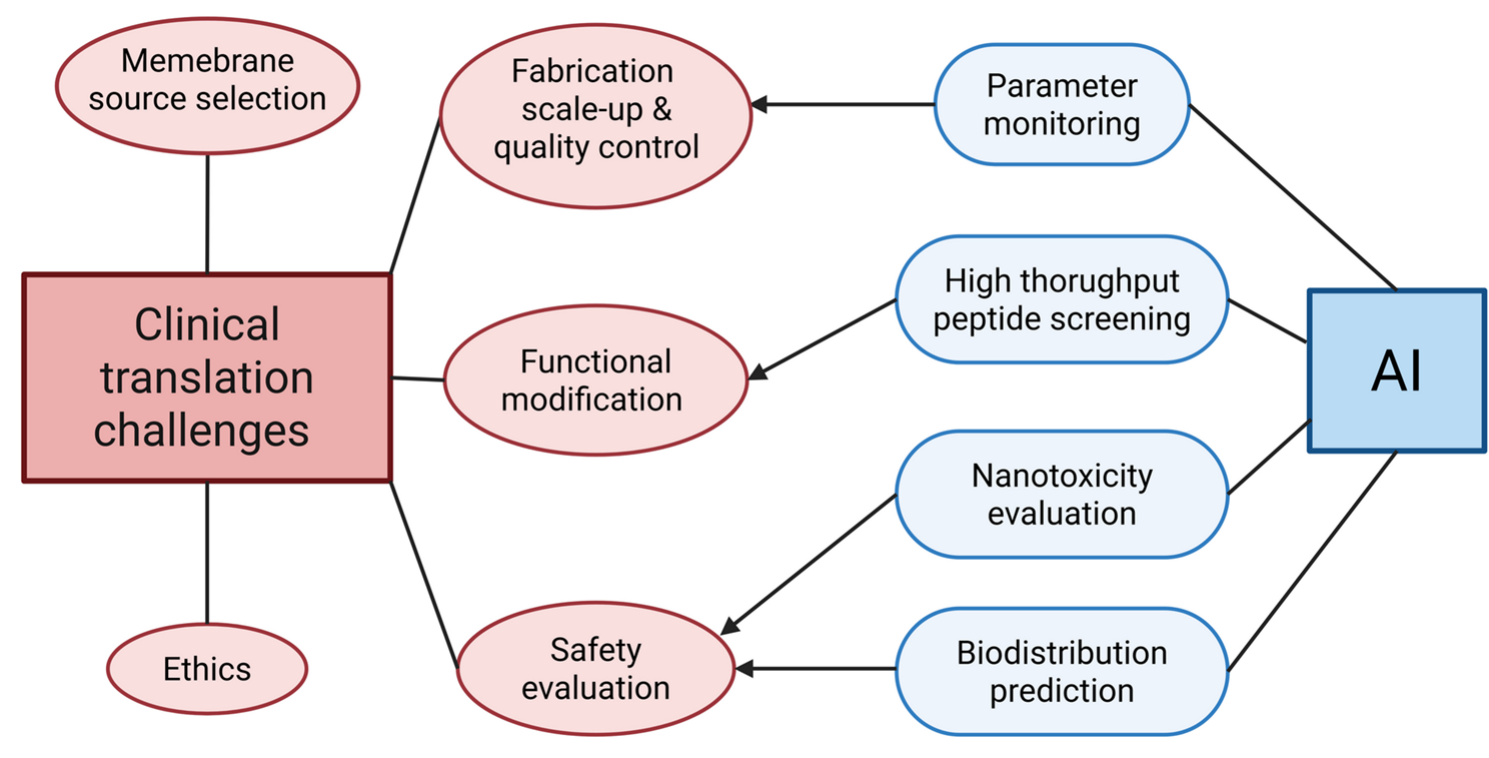
Artificial Intelligence (AI)-assisted strategies addressing clinical translation challenges of BBB-crossing CNPs. AI facilitates the efficient development of CNS-targeted CNPs by enabling parameter monitoring, peptide screening, nanotoxicity evaluation, and biodistribution prediction

Collectively, artificial intelligence holds transformative potential for advancing rational design, property characterization, and safety prediction of CNPs for the clinical translation of CNS-targeted drug delivery platforms (Fig. 9). However, implementation is currently limited by both a lack of comprehensive datasets and an absence of key descriptors predictive of membrane function. Much like for reliable manufacturing, fully utilizing the potential of AI for reliable prediction and design of CNP properties will require the development of new experimental techniques to comprehensively characterize CNPS (size, zeta potential, protein and lipid composition of the membrane) on a rapid batch-by-batch basis. For optimization of BBB crossing specifically, new assays may be needed to quickly evaluate CNP flux through the various interstitial and transcytotic pathways to enable future AI models to accelerate the development of mechanistic insights in these areas. Although most current applications remain centered on conventional nanoparticles, the extension of these AI-driven methodologies to CNPs, through an interdisciplinary effort to improve understanding of membrane-specific features and functional parameters, holds promise to overcome key challenges in crossing the BBB. Continued innovation at the interface of nanotechnology and AI will be critical for realizing the full potential of CNPs in treating CNS disorders.

## Conclusion

This review summarizes current BBB-crossing strategies, with a particular focus on emerging biomimetic nanoplatform—cell membrane-engineered nanoparticles (CNPs)—which leverage the intrinsic properties of source cell membranes to enhance therapeutic efficacy. Preclinical biosafety evaluations have further supported the low toxicity of CNPs compared to unmodified nanoparticles. Although cell membrane coating represents a promising and versatile approach for CNS-targeted drug delivery, continued efforts toward improving functional modifications, establishing reliable large-scale production protocols, and generating robust long-term safety data will be essential for advancing CNPs toward clinical translation in the treatment of neurological diseases.

## Data Availability

No datasets were generated or analysed during the current study.

## Abbreviations

AD: Alzheimer’s Disease
AI: Artificial Intelligence
AIE: Aggregation-Induced Emission
ALT: Alanine Transaminase
ALP: Alkaline Phosphatase
AST: Aspartate Transaminase
BBB: Blood–Brain Barrier
Bcl-2: B-cell Lymphoma 2
CBD: Cannabidiol
CCNP: Cancer Cell Membrane-Engineered Nanoparticle
CNP: Cell Membrane-Engineered Nanoparticle
CNS: Central Nervous System
Dox: Doxorubicin
DTX: Docetaxel
FDA: Food and Drug Administration
FNM: Iron–Nitrogen-Doped Mesoporous Carbon
ICG: Indocyanine Green
ICAM-1: Intercellular Adhesion Molecule 1
LA: α-Lipoic Acid
Lex: Lexican
LFA-1: Lymphocyte Function-Associated Antigen 1
LOX: Lactate Oxidase
LRP1: Low-Density Lipoprotein Receptor-Related Protein 1
Mac-1: Macrophage-1 Antigen
MCNP: Macrophage Membrane-Engineered Nanoparticle
NCNP: Neutrophil Membrane-Engineered Nanoparticle
NK: Natural Killer
NKCNP: Natural Killer Cell Membrane-Engineered Nanoparticle
NPs: Nanoparticles
nAChRs: Nicotinic Acetylcholine Receptors
NIR: Near-Infrared Irradiation
PCNP: Platelet Membrane-Coated Nanoparticle
PEG: Polyethylene Glycol
PTT: Photothermal Therapy
QS: Quorum-Sensing
RBCNP: Red Blood Cell Membrane-Engineered Nanoparticle
ROS: Reactive Oxygen Species
RSV: Resveratrol
saRNA: Small Activating RNA
SIRP-α: Signal Regulatory Protein Alpha
TfR: Transferrin Receptor
TJ: Tight Junction
TM: Temozolomide
UA: Uric Acid
VCAM-1: Vascular Cell Adhesion Molecule 1
ZO-1: Zonula Occludens-1
